# 10-Hz tACS over the prefrontal cortex improves phonemic fluency in healthy individuals

**DOI:** 10.1038/s41598-022-11961-8

**Published:** 2022-05-18

**Authors:** Ya Sun, Lihui Huang, Qiaoling Hua, Qiang Liu

**Affiliations:** 1 Institute of Brain and Psychological Sciences, Sichuan Normal University, Chengdu, 610066 China; 2 Faculty of Education, Sichuan Normal University, Chengdu, 610066 China; 3 Research Center of Brain and Cognitive Neuroscience, Liaoning Normal University, Dalian, 116029 China

**Keywords:** Psychology, Human behaviour

## Abstract

Verbal fluency is an important indicator of human verbal ability. Methods to improve fluency is an interesting issue necessitating investigation. To do this, the current study required participants to randomly receive transcranial alternating current stimulation (tACS) at 10 Hz, 40 Hz (control frequency), and sham stimulation over the prefrontal cortex before a phonemic fluency task. It was found that 10-Hz tACS significantly improved phonemic fluency relative to sham stimulation. This result demonstrates the modulatory effect of 10-Hz tACS on language ability.

## Introduction

Verbal fluency is a cognitive function that facilitates the retrieval of knowledge in memory. It describes the efficiency of individuals’ vocabulary production, and it is an important indicator of human verbal ability. Phonemic fluency mainly related to letters is one important indicator of verbal fluency^1^. Generally, a verbal fluency task requires subjects to say words according to certain rules in limited time. Importantly, a phonemic fluency task is variable and depends on the language. In the English version, subjects are often asked to start with a specific letter, such as the letters ‘f’, ‘h’, etc., to generate words^2^. In the Chinese version, on the other hand, some studies require subjects to output as many words as possible starting with a certain letter^3^ or an initial consonant^4^ within a fixed time. The effect of different languages on phonemic fluency is different. Some studies have found different activation patterns between languages in verbal fluency tasks. For example, it has been noted that Italian has stronger activation in the supratemporal gyrus than English^5^. Chinese, as a typical ideographic script, differs from phonetic scripts in terms of orthography and phonological rules. In phonemic fluency tasks, phonetic scripts generally require subjects to generate vocabulary with a initial letter , while the relationship between pronunciation and letters in phonetic scripts is so close that there are not only phonological but also graphemic cues. When the initial letter is fixed, subjects can associate words from the glyphs of the initial letter. In contrast, Chinese pronunciation is generally not reflected in graphemes, the relationship between Chinese phonology and graphemes is not so strong, and Chinese phonemic positions are relatively more independent. There are no graphemic cues in the phonemic verbal fluency task of Chinese. Therefore, the phonological processing method in Chinese is different from that of phonetic scripts, and the phonemic fluency task in Chinese is more difficult and may involve more complex cognitive processes. For example, the existence of Chinese-specific brain activation patterns has been demonstrated^6^. Therefore, it is essential to study Chinese verbal fluency.

Increasing evidence supports that the left frontal lobe plays a crucial role in phonemic fluency. A positron emission tomography study found that phonemic fluency elicited more activation in the left frontal lobe than other areas^7^. Similarly, phonemic fluency has been shown to activate the left inferior frontal gyrus in functional magnetic resonance studies^8^. Brain injury studies provide direct evidence that phonemic fluency performance decreased when the left frontal lobe was impaired^9^. In addition, many tDCS studies have shown that anodal stimulation targeting the left frontal lobe increased the number of produced words, while cathodal stimulation decreased the number of words^10–12^. A study on repetitive transcranial magnetic stimulation (rTMS) also showed that the use of hf (high frequency)-TMS in the left frontal lobe improved patients' verbal fluency^13^. Studies have shown that semantic fluency tests lack the usefulness of identifying key lesions compared to phonemic fluency, as all patients (left and right frontal lobe, temporal lobe, etc.) show equal deficits in semantic fluency, while phonemic fluency is sensitive and specific to frontal lobe damage^9^. Altogether, these findings suggest that phonemic fluency is sensitive and specific to the left frontal lobe.

In previous studies on verbal fluency, transcranial direct current stimulation techniques have received a lot of attention and application from researchers, while current studies have shown that transcranial alternating current stimulation techniques are more effective than transcranial direct current stimulation techniques in some areas of cognitive function. For example, in a study on associative memory found that tACS may be more effective than tDCS in improving associative memory performance in older adults^14^. Also, in a study on working memory directly comparing the effects of theta tACS (6 Hz) and anodal tDCS on working memory, it was found that tACS was significantly better than tDCS for improving working memory^15^. Recently, transcranial alternating current stimulation (tACS) offered a new promising method to improve phonemic fluency^16–18^. tACS applies periodic alternating weak currents to specific brain regions, delivering alternating currents of specific frequencies between electrodes in a bidirectional manner^16,19^. Previous studies have shown tACS improves language function. For example, one study found that 10-Hz tACS acting on the left prefrontal cortex significantly accelerated language responses in healthy individuals^20^; whereas, Katharina observed that 40-Hz tACS reduced the ability of phonological categorisation in young adults but enhanced such ability in the elderly^21^. In addition, it has been shown on the bilateral prefrontal cortex that tACS regulates creative thinking in the language domain^22^. However, the effect of tACS on phonemic fluency remains unknown.

To this end, the current study selected 10-Hz, 40-Hz, and sham stimulation over the left frontal lobe to investigate the influence of different frequencies of tACS on verbal fluency. Since 10-Hz and 40-Hz tACS will alter different cortical excitatory activities, we hypothesize that tACS modulates verbal fluency and that for healthy individuals, tACS at 10 Hz will increases phonemic fluency and tACS at 40 Hz will decreases phonemic fluency.

## Materials and methods

### Participants

In the study, 18 healthy, Chinese native speakers (9 female, 9 male) aged 18–25 years (*M* = 20.11, *SD* = 3.36) participated in the study. To calculate our sample size, we used g*Power with the following settings: effect size = 0.25, a level = 0.05, power = 0.9, and correlation among repeated measures = 0.7. The minimum sample size was found to be 16, which we increased to 18 to fully counterbalance the order of stimulation conditions across participants. All participants were right-handed according to the Edinburgh Handedness Inventory. None of them took any medication, had a history of neurological diseases, had metallic head implants, or had stuttering problems. To avoid practice effects, the same subjects did not participate in both the phonemic fluency materials and the formal experiment.

### Phonemic fluency materials

Phonemic fluency tests are widely used in language research^23,24^, so the present study also employed the task and did a phonemic fluency materials to balance the difficulty of the experimental material across stimulation sessions. There were 24 subjects (12 males, 12 females) with an average age of 24 years who did not participate in the formal experiment but completed the phonemic fluency materials.

In the study of Chinese verbal fluency, it is controversial whether to use a ‘consonant’ as a measure of phonemic fluency, or to follow the phonetic alphabet to use initial letter as a measure of phonemic fluency. As we mentioned above Chinese differs from phonetic scripts in terms of orthography and phonetic rules. It is obviously unreasonable to follow the rules of phonetic alphabet^25^. Since the relationship between Chinese pronunciation and graphemes is not as close as that of phonetic scripts, we believe that the "consonant" indicator is a more sensitive measure of Chinese phonemic fluency and a more appropriate localized measurement. In current studies related to Chinese phonemic fluency, the "consonant" task is often used to examine phonemic fluency^6^. Therefore, the present study required participants to produce as many words as possible starting with a specific consonant in one minute. Based on the number of words in the lexicon, we selected the consonants ‘j’, ‘d’, ‘x’, ‘y’, ‘sh’, ‘zh’, ‘b’, and ‘m’ as the initial phonemes. The exceptions were proper nouns, such as names of people and places, or words that were repeated.

According to the phonemic fluency materials, we excluded the consonant ‘b’, which produced the largest number of words, and the consonant ‘m’, which produced the least number words. The remaining consonants of ‘y’, ‘j’, and ‘sh’ were divided into difficulty-balanced group A, and the consonants ‘d’, ‘x’, and ‘zh’ were divided into difficulty-balanced group B. Each fluency task included one Group A consonant and one Group B consonant, counterbalancing the difficulty of stimulation sessions.

### Control tasks

To reveal whether the effect of tACS on phonemic fluency depended on changed attention or the subjects’ expectations, each subject had to implement a control task after the phonemic fluency task. In the control task, a gaze point or an arrow appeared in the middle of the screen, and the arrow randomly directed to the left or right. Subjects had to determine the direction of the arrow by pressing a key as quickly as possible. The reaction time and accuracy were then recorded. There were 200 trials in total, with an interval of 800 ms.

### tACS protocol

In the present study we focused on the aftereffects of tACS rather than its online effects. It was shown that the aftereffect of tACS may be different from the online effect during stimulation, the online effect of tACS is a fixation activity, whereas the aftereffect of tACS reflects short-term synaptic plasticity in response to stimulation^26^. Furthermore, only a few studies have combined behavioral and tACS aftereffects, but a better understanding of the tACS aftereffects is necessary for the technique as well as its clinical treatment. Therefore, in the present study we chose to focus on the after-effects of tACS.

The impedances were kept below 10 kΩ. We applied oscillating currents at 10 Hz, 40 Hz, or sham stimulation for 20 min. The current was ramped up and down over the first and last 15 s of stimulation. During sham stimulation, the current was ramped up for 15 s, followed by 30 s of 1 mA stimulation (at either10 Hz or 40 Hz) and then ramped down for 15 s. Prior to the experiment, all subjects underwent a tACS threshold measurement to determine the individual threshold. For this purpose, we used a tACS of 1.0 mA and gradually increased or decreased the stimulation by 0.1 mA. Subjects were asked to keep their eyes open and were asked about any visual or sensory changes. To ensure that the participants did not feel the stimulation but were stimulated throughout the experiment, the stimulation intensity for the formal experiment was kept at 0.1 mA below the lower limit of skin sensation. This approach allowed for adequate stimulation based on individual physiological parameters and also ensured that subjects were unable to distinguish between the three stimulations. tACS-intensity was adjusted below individual phosphene- and discomfort threshold and ranged from 1.2 to 2 mA. The order of the three tACS stimulations was counterbalanced across all subjects.

### Experimental procedure

**Figure 1:**
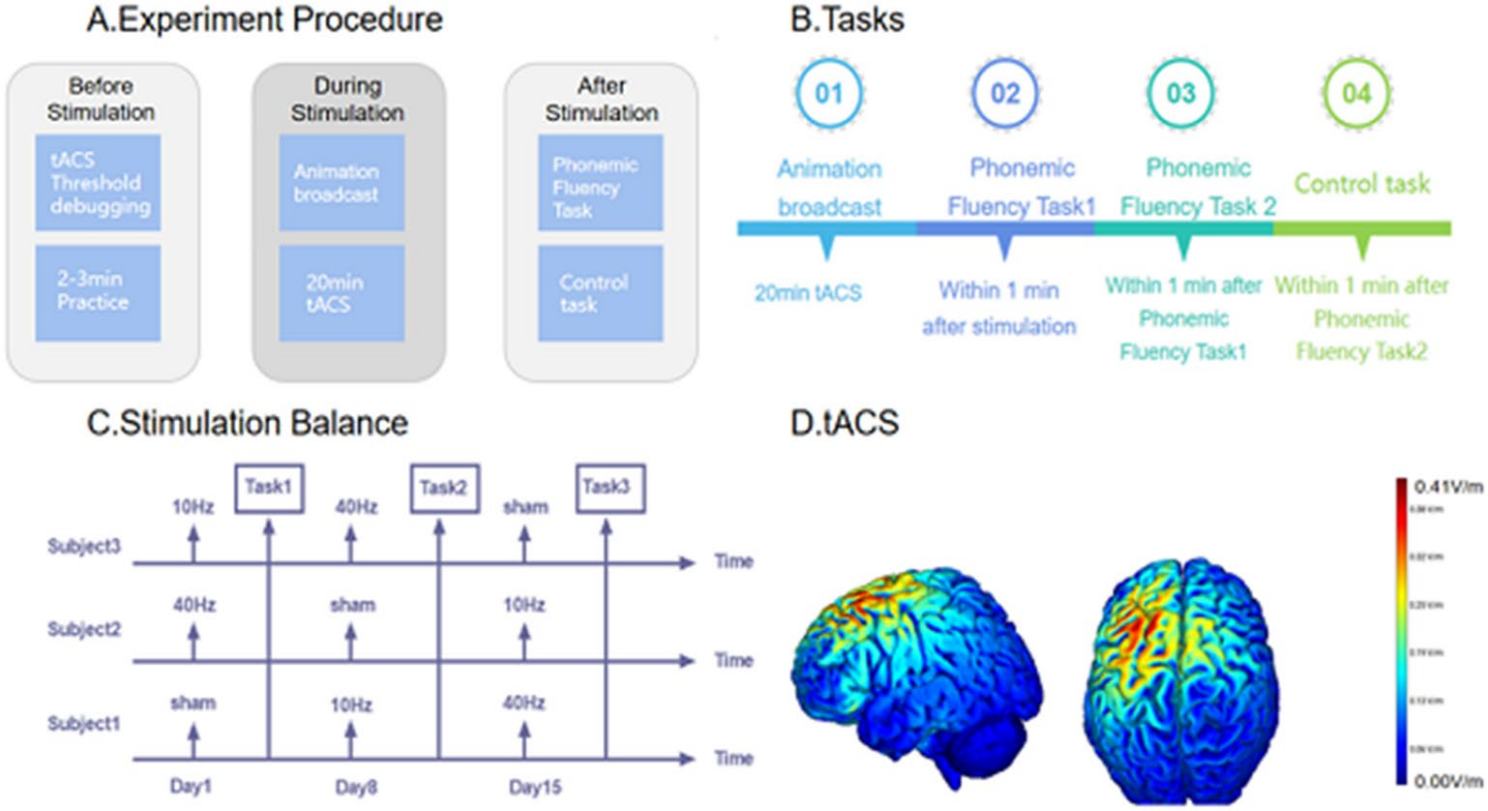
Experimental design. (**A**) Time-course of the experiment. At the beginning of each session, we completed a tACS threshold debugging, participants performed a short training of the phonemic fluency task. After 20 min of tACS, subjects performed two phonemic fluency tasks and a control task (**B**) Tasks. (1) Phonemic fluency task. Requires subjects to say words according to a certain consonant limited in 1 min. (2) Control task. Directional arrows were presented visually and subjects had to indicate their direction by pressing the corresponding button. (**C**) The order of stimulations. Each subject will be given three stimuli in three sessions.Each stimulus has an interval of 7 days. (**D**) Simulation of the electric field of the stimulation.

The specific experimental procedure is presented in Fig. 1. In a normally lit and quiet room, the subject sat in front of a computer screen. Before the experiment, each subject would practice for 2–3 min using consonants that would not appear in the experiment until they were familiarised with the task. Subjects were then asked to watch a cartoon and were given tACS stimulation at the same time. All subjects watched the same silent cartoon, both during the real stimulus and the sham stimulus. Note, to reduce the variability between subjects with the same visual experience, all subjects watched the same cartoon in all three stimulation sessions. After 20 min of tACS stimulation, participants completed two separate phonemic fluency tasks. The output words would be recorded via the computer recording device.

**Figure 2:**
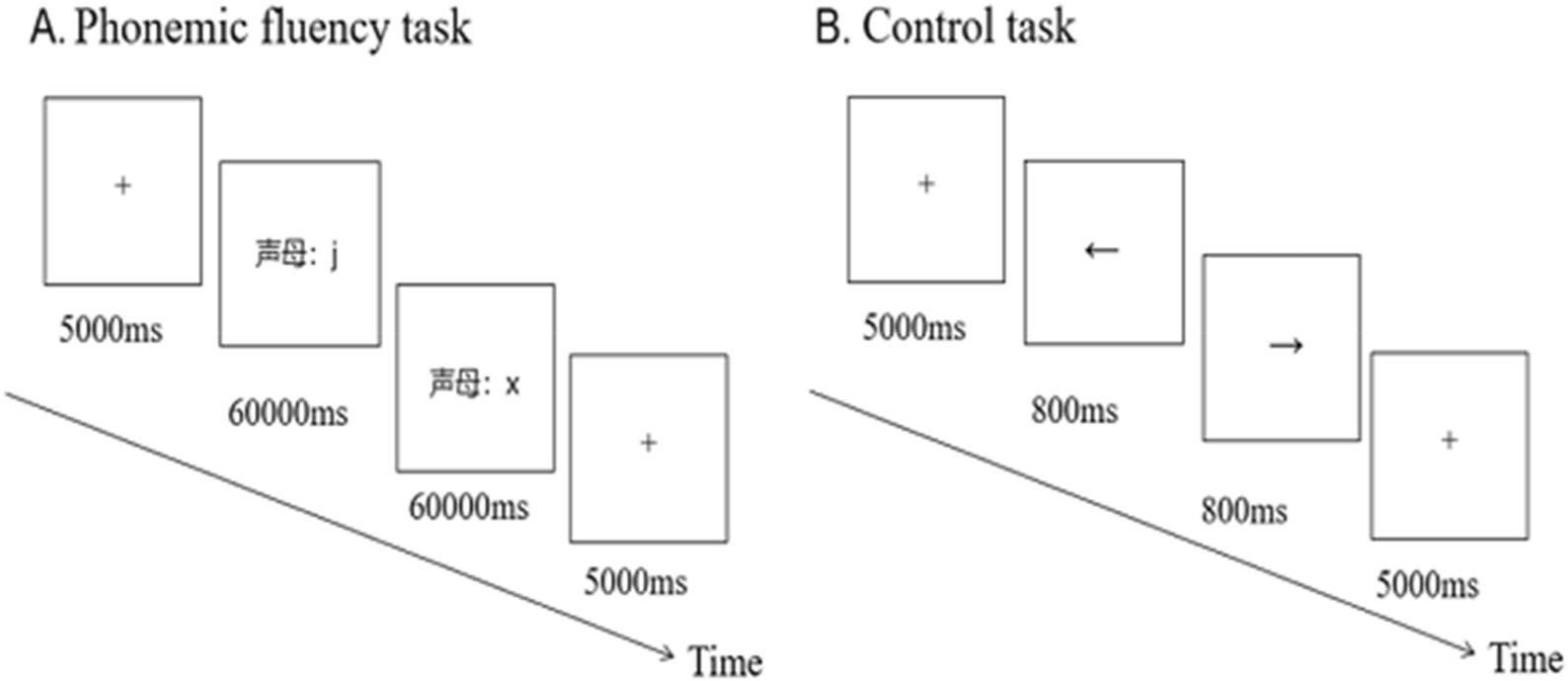
Tasks procedure. (**A**) Phonemic fluency task. In the phonemic fluency task, stimuli were presented visually for 60,000 ms, the words produced by the subjects were recorded via a recording device. (**B**) Control task. Directional arrows were presented visually and subjects had to indicate their direction by pressing the corresponding button.

Since a 20-min tACS aftereffect can last from 30 to 60 min^16^, whereas our formal experiment and control tasks lasted only 2–3 min, it was ensured that the subjects throughout the formal experimental and control tasks had a similar tACS effect. Therefore, at the end of each stimulation session, participants performed a control task. A single-blind design was adopted for this study. The matches of three sets of stimulation (10-Hz tACS, 40-Hz tACS, and sham tACS) and the phonemic fluency task were counterbalanced across participants. Each stimulus has an interval of 7 days. For example, subject A received a 10-Hz tACS stimulation on day 1 and completed a phonemic fluency task. Seven days later, subject A received sham tACS stimulation and completed a second phonemic fluency task. After another 7 days, a 40-Hz tACS stimulation was administered, followed by a final phonemic fluency task. The stimulation used in the three phonemic fluency tasks was different and counterbalanced across participants. In addition, to avoid a practice effect, the order of stimulation frequencies was also counterbalanced across participants. The specific program diagram is presented in Fig. 2.

### Ethical approval

The study was conducted according to the guidelines of the Declaration of Helsinki and approved by the Ethics Committee of the Institute of Brain and Psychological Sciences, Sichuan Normal University (the approval number: SICNUIRB200608).

### Informed consent

Informed consent was obtained from all participants involved in the study.

## Results

The indices of the phonemic fluency task included (1) the number of produced words excluding incorrect, repeated words, and nonsense words and (2) the reaction time when the subject reaches the fifth word. Statistical analysis was performed using SPSS 16.0 software. The number of produced words and the time when reaching the fifth word were submitted to a two-way repeated-measures ANOVA crossing stimulation session (10 Hz, sham stimulation, 40 Hz).

### Phonemic fluency materials

**Figure 3:**
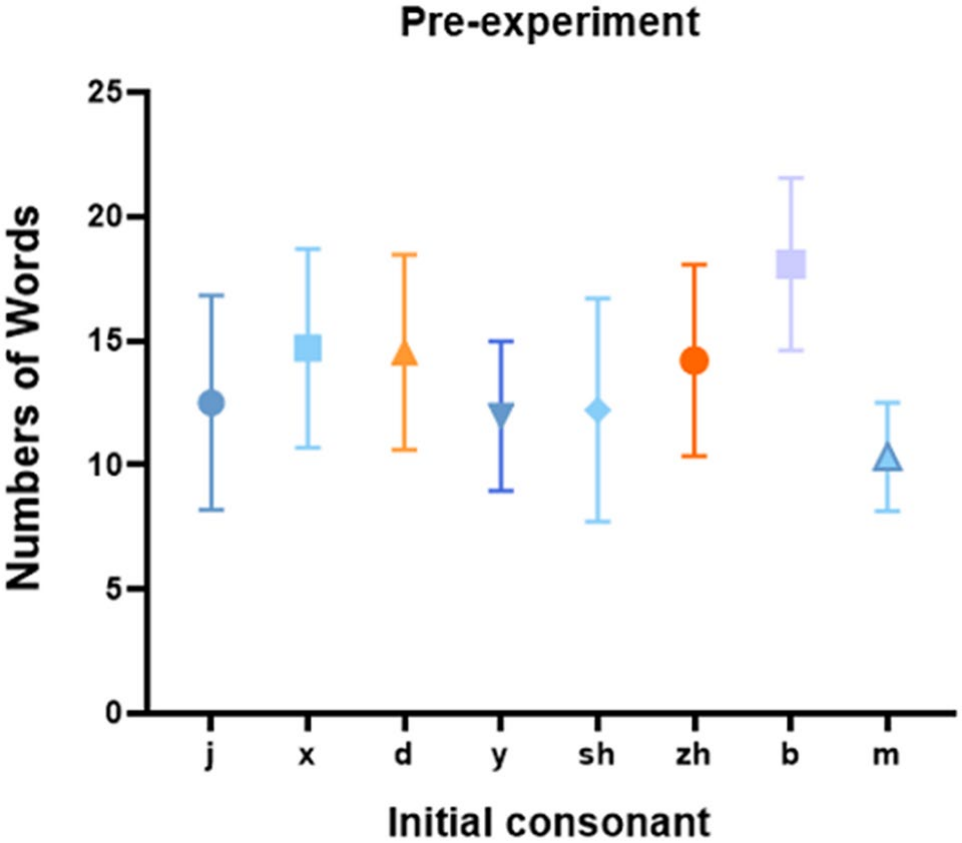
Phonemic fluency material results. The mean number of words for each consonant in the pilot experiment.

We analysed the number of words produced in the phonemic fluency materials. After excluding errors and repetitions, Fig. 3 shows the words produced by the subjects in the phonemic fluency materials for each consonant condition. Based on the average vocabulary produced, we tentatively classified the consonants ‘x’, ‘d’, ‘zh’, and ‘b’, which difficulty-balanced to the A group. The consonants ‘j’, ‘y’, ‘sh’, and ‘m’, which difficulty-balanced, were divided into the B group. The results of the ANOVA for the A group were *F*(1,23) = 9.902 *p* = 0.001, *n*^2^ = 0.29, which is a significant difference in difficulty. After removing the consonant ‘b’, we did not find a significant main effect of difficulty, *F*(1,23) = 0.150, *p* = 0.861, *n*^2^ = 0.76, indicating no difference in difficulty in the produced words using these consonants. Therefore, we removed the consonant ‘b’ and divided the consonants ‘x’, ‘d’, and ‘zh’ into one group. As for the B group, the main effect of the difficulty reached significance, *F*(1,23) = 4.243 *p* = 0.008, *n*^2^ = 0.83. After removing the consonant ‘m’, the main effect of difficulty was not significant, *F*(1,23) = 0.297, *p* = 0.744, *n*^2^ = 0.13. Therefore, we removed the consonant ‘m’ and divided the consonants ‘j’, ‘y’, and ‘sh’ into one group. The specific phonemic fluency materials data is presented in Table 1, and the specific phonemic fluency materials results is presented in Fig. 3.

**Table 1:** Number of words generated in the pre-experiment.

Consonant	j	d	x	y	sh	zh	b	m
Mean numbers	12.5	14.5	14.7	11.9	12.2	14.2	18.08	10.3
SD	4.31	3.9	3.99	3.01	4.48	3.32	3.46	2.18

### Phonemic fluency task

**Figure 4:**
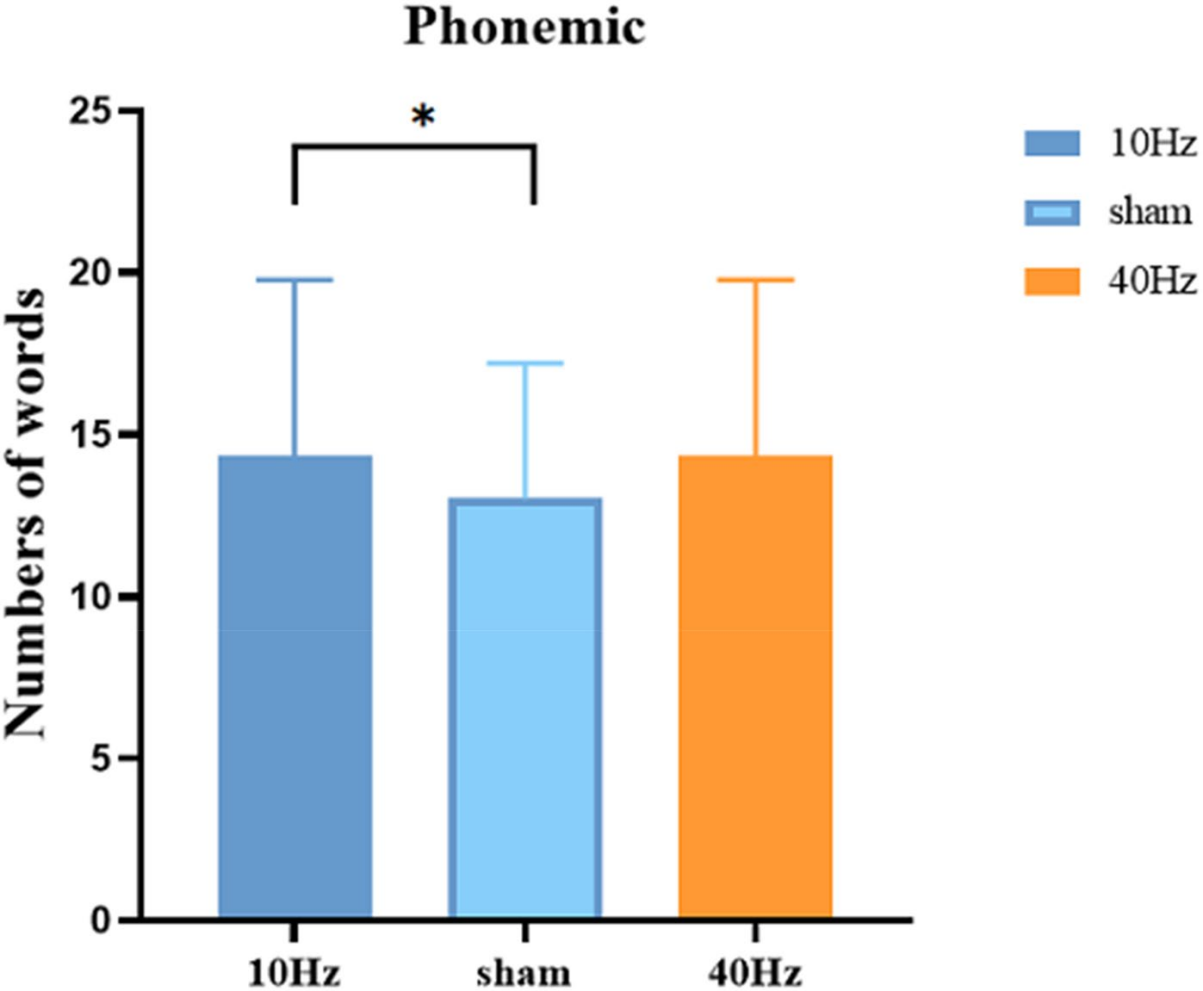
Phonemic fluency task results graph. The mean number of words for each consonant produced in the phonemic fluency task.

We analysed the average number of words produced. After excluding errors and repetitive words, Table 2 shows the mean number of words produced by the subjects in the phonemic fluency task in 10-Hz tACS, sham stimulation, and 40-Hz tACS, respectively. The interaction between the task and stimulation was analysed and showed a significant main effect of stimulation, *F*(2,34) = 5.636, *p* = 0.008, *n*^2^ = 0.16, such that 10-Hz tACS significantly increased the amount of vocabulary produced by the subjects in the phonemic fluency task compared to the sham stimulation (Fig. 4). The main effect of task was not significant, *F*(2,34) = 0.949, *p* = 0.344, *n*^2^ = 0.86. The stimulation-task interaction was not significant, *F*(2,34) = 0.432, *p* = 0.653, *n*^2^ = 0.32. The result was *F*(2,34) = 3.807, *p* = 0.032, *n*^2^ = 0.183. The number of words produced by phonemic fluency is presented in Table 2, and the mean number of words for each consonant produced in the phonemic fluency is presented in Fig. 4.

**Table 2:** Number of words produced by phonemic fluency.

Stimulation	10 Hz	Sham	40 Hz
Mean numbers	15.25	13.02	14.36
SD	5.38	4.17	5.42

### The reaction times required to reach the fifth word

**Figure 5:**
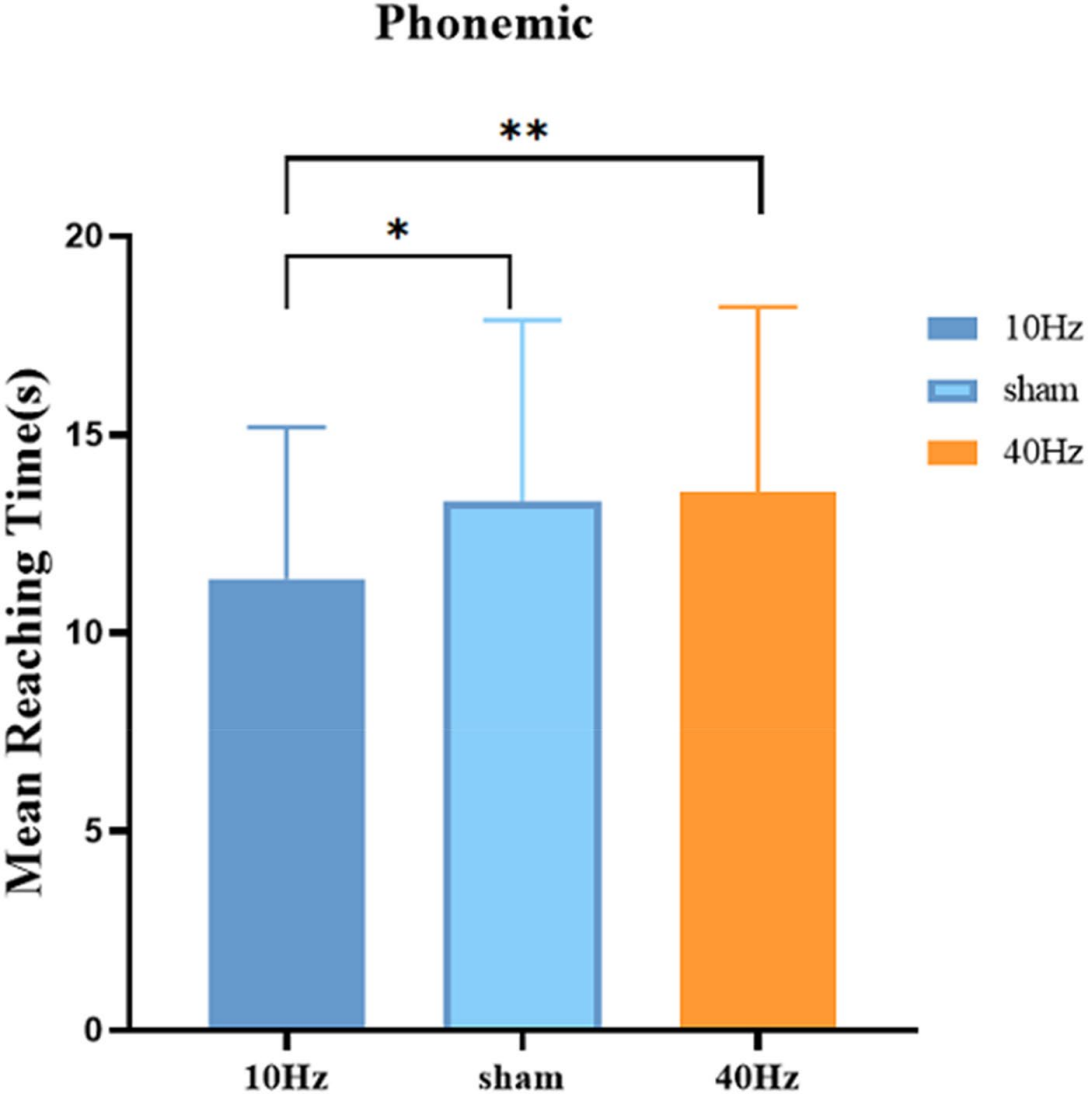
The time required for subjects to reach the fifth word. Mean reaching time (average for one consonant) in the phonemic fluency task in the experiment.

Table 3 shows the time required for subjects to reach the fifth word. We analysed the reaction times required to reach the fifth word. After excluding errors and repetitive words. A repeated-measures ANOVA obtained a significant main effect of stimulation session, *F*(2,34) = 3.985, *p* = 0.039, *n*^2^ = 0.15. As shown in Fig. 5, compared to the sham stimulation and 40-Hz, 10-Hz tACS significantly increased time until the subjects reached the fifth word. The interaction between the task and stimulation session was not significant, *F*(2,34) = 0.766, *p* = 0.473, *n*^2^ = 0.07.

**Table 3:** Time to reach the fifth word.

Stimulation	10 Hz	Sham	40 Hz
Mean reaching time	11.36	13.30	13.52
SD	3.81	4.57	4.67

### Control experiment

**Figure 6:**
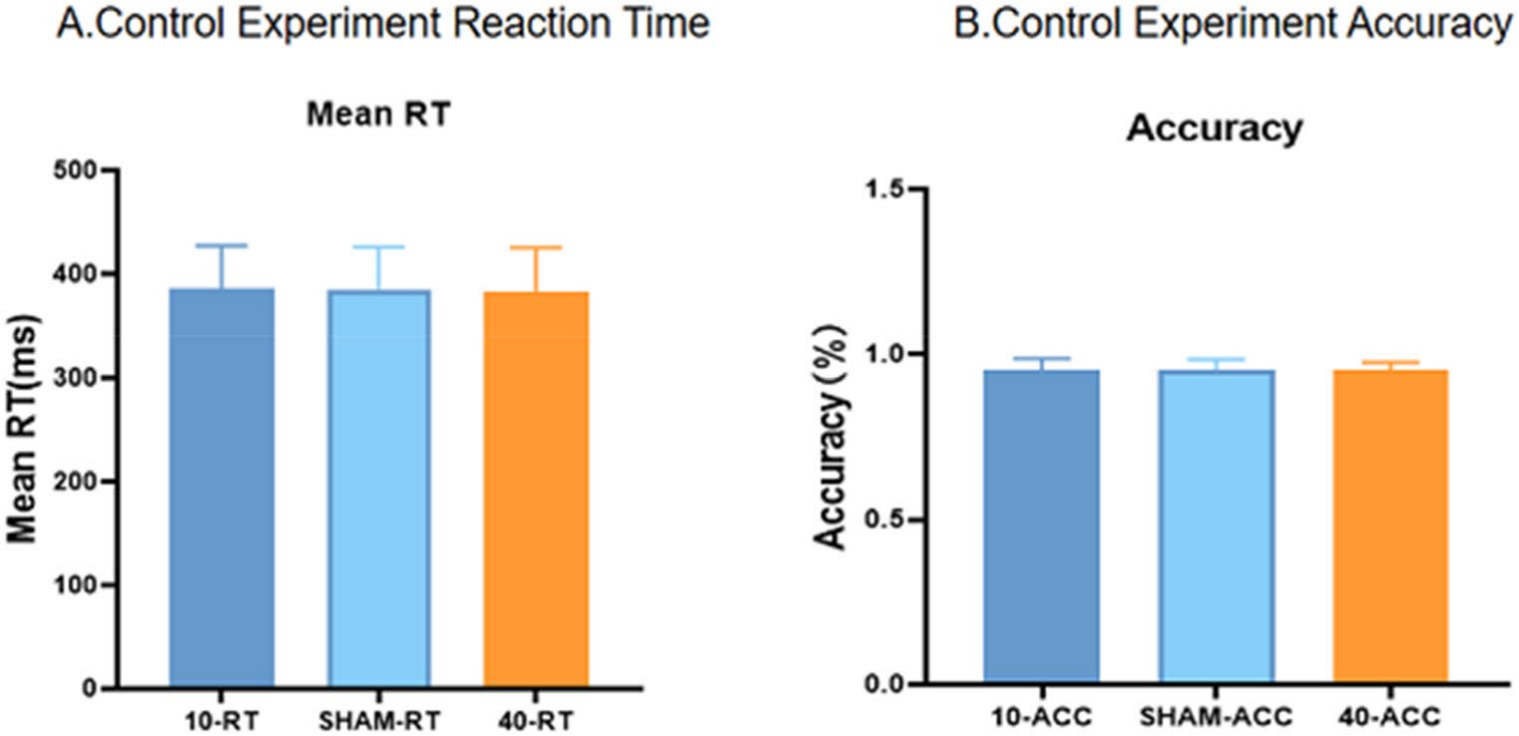
Control experiment results. (**A**) Control experiment reaction time. (**B**) Control experiment accuracy.

The reaction time and correct rates of the subjects in the control experiment are shown in Fig. 6. A repeated-measures ANOVA was conducted for the response times in the control experiment for the three stimulation conditions (10-Hz tACS, sham stimulation, and 40-Hz tACS). The results were *F*(2,34) = 0.131, *p* = 0.878, *n*^2^ = 0.72. All three stimulations did not significantly speed up or slow down the reaction time. In the same way, the accuracy in the control experiment for the three stimulation conditions did not differ, *F*(2,34) = 1.482, *P* = 0.243, *n*^2^ = 0.63.

These findings indicate that the effect of tACS on phonemic fluency was not dependent on the effect of tACS on the subjects' attention and was not influenced by the subjects' expectations.

## Discussion

In the present study, we respectively induced 10-Hz tACS, 40-Hz tACS, and sham stimulation over the prefrontal cortex to investigate the effect of tACS on phonemic fluency. Compared to the sham stimulation, 10-Hz tACS significantly increased the amount of vocabulary produced by the subjects in the phonemic fluency task. Also, the response time to reach the fifth word in the phonemic fluency task was significantly shorter with 10-Hz tACS compared to the sham stimulation and 40-Hz tACS. This suggests that 10-Hz tACS improved subjects' response time in the phonemic fluency task.

It is noteworthy that the improvement in phonemic fluency with 10-Hz tACS complements previous studies on tACS in relation to language. According to previous studies, 10 Hz-tACS over the prefrontal cortex significantly facilitated speech response speed in healthy individuals^20^. In that study, it was reported that 10-Hz tACS significantly increased theta power during voice decision-making. This may indicate that the improvement in phonemic fluency may be associated with increased theta power. In a study about tACS and fluid intelligence, it was found that the tACS aftereffect of fluid intelligence was associated with changes in theta and alpha bands, which may have functional cross-frequency modulation^27^; furthermore, theta tACS improved subjects' performance on fluid intelligence tests^28^. Previous studies have confirmed that theta is associated with the processing of linguistic features^21^. We presume that this may explain why 10-Hz tACS improves phonemic fluency in healthy individuals. Several studies have suggested that verbal fluency is supported by fluid intelligence, meaning that an individual's fluid intelligence affects their verbal fluency. For example, in a large-scale study of frontal lobe patients, researchers interpreted the manifestation of impaired verbal fluency as resulting from a deficit in flow intelligence, which is thought to reflect current abstract thinking and reasoning abilities^29^.

Previous studies have found effects of tACS on working memory and visuomotor perception as well as other factors. For example, in one study it was shown that tACS applied to the frontal regions significantly improved subjects' working memory capacity^30^. Likewise, in a study on tACS and motor function and motor cortical excitability, tACS was shown to have a particularly pronounced effect on motor variability^31^. Thus, the current behavioural aftereffects may also be due to the effects of tACS on working memory or other factors. However, our control task showed that subjects' reactivity and attention were not affected by different tACS frequencies (either true or false stimuli) or by different tasks. Therefore, the possibility that the improvement of phonemic fluency by tACS may be indirectly due to the improvement of other cognitive functions may be excluded.

In the 40-Hz tACS stimulus, due to our current experiments, we have not found any significant effect of stimulants on performance yet, either on the amount of words produced or on the time to reach the fifth word. This result contrasts with our experimental hypothesis based on the study by Katharina^21^ who found that 40-Hz tACS reduced phonemic categorisation ability in young people and improved phonemic categorisation ability in older people. Although phonemic categorisation ability is relevant to language ability, it is primarily related to perceptual learning and the auditory system, whereas 40-Hz tACS impairs perceptual learning by interfering with relevant neurotransmitters in the auditory system. However, phonemic fluency and phonological categorisation ability depend on different mechanisms, so 40-Hz tACS had no effect in our study. In the current study, only a few studies have used the aftereffects of tACS to study language, so the aftereffects of tACS on language remain to be discussed.

Many tACS-related studies are also currently demonstrating the promise of tACS technology as a clinical tool used to modulate brain activity in the treatment of various neurodegenerative diseases. In studies related to tACS and Alzheimer's disease, tACS was found to improve autobiographical memory^32^ and cognitive function^33^. tACS was found to be effective in both motor and cognitive symptoms in Parkinson's disease patients^34^, and it was useful for conditions such as schizophrenia and ADHD^35^. There is also current evidence supporting a neuroprotective (i.e. disease-modifying) role of tDCS/tACS in neurodegenerative diseases^36^. Based on the relationship between tACS and phonemic fluency found in this study, we believe that tACS may have great potential in the clinical treatment of disorders such as aphasia, and we can expect a greater role for tACS technology in aphasia and disorders caused by disorders of verbal expression in the future.

## Limitations and future directions

The limitation of this study was the lack of comparison of the online effect of tACS with the offline effect. Although we have mentioned above that the effects are not the same between online effect and offline effect, this issue needs to be further investigated as there are no studies combining online tACS with verbal fluency. The lack of individualized brain MRI scans and electroencephalography (EEG) was also a limitation of this study. Future analysis of EEG data and follow-up studies with more targeted neuroimaging are needed to explore changes in functional connectivity to facilitate utility in clinical care. Finally, we did not consider in our study the possibility of stimulating areas outside the prefrontal cortex that are likely not involved in the study of phonemic fluency. Stimulation of these areas was compared with prefrontal areas to explore whether participants distinguished between true and false stimuli and whether the improvement in verbal fluency by tACS was caused by specific brain regions. As done in Experiment 2 by Cattaneo et al.^2^. Therefore, in future studies, we need to explore study protocols more suitable for tACS, choosing online or offline effects, and comparing more different brain regions. Due to the close relationship between tACS and neural oscillations, further research will need to combine brain imaging devices and tACS.

## Conclusions

In summary, our results found for the first time that 10-Hz tACS over the prefrontal cortex significantly improved phonemic fluency in healthy individuals, including the number of words generated as well as response speed. Our data confirm the modulatory role of tACS for language ability and its potential role in neurological rehabilitation, and further point to the frequency specificity of tACS. In future studies on tACS and phonemic fluency, the differences brought by different languages, the clinical application of tACS in conditions such as aphasia, and the application to the improvement of phonemic fluency in healthy individuals should be emphasised.

## Data Availability

Data are available on request due to restrictions (for example, privacy or ethical). The data presented in this study are available on request from the corresponding authors.
